# Bupropion Toxicity Presenting As Alcohol Withdrawal Syndrome: A Case Report

**DOI:** 10.7759/cureus.106899

**Published:** 2026-04-12

**Authors:** Darwin Nguyen, Daniel Faradji, Alex Frangenberg, Saba Haq

**Affiliations:** 1 Internal Medicine, Methodist Dallas Medical Center, Dallas, USA

**Keywords:** agitation, alcohol withdrawal syndrome, bupropion toxicity, diagnostic errors, drug overdose, encephalopathy, hydroxybupropion, seizures

## Abstract

Bupropion toxicity can present as neurologic and autonomic manifestations that closely resemble alcohol withdrawal syndrome, a diagnostic challenge that becomes compounded when patients are unable to provide reliable histories and when confirmatory toxicology results are delayed. We present the case of a 55-year-old male who presented with agitation, encephalopathy, auditory and visual hallucinations, hypertension, tremors, and suspected seizure activity. Based on his substance use history and clinical presentation, he was managed as alcohol withdrawal using symptom-triggered benzodiazepines per the Clinical Institute Withdrawal Assessment for Alcohol-Revised protocol. Serum ethanol level and urine toxicology screen were negative on admission, and he improved with supportive care and benzodiazepine therapy in the intensive care unit. Serum toxicology results returned after discharge and revealed markedly elevated bupropion and hydroxybupropion concentrations, establishing clinically significant bupropion toxicity as the true etiology. While bupropion toxicity mimicking alcohol withdrawal has been described, cases in which the diagnosis was established only after discharge, when quantitative metabolite assays returned, remain rare. This case illustrates how convincingly bupropion toxicity can masquerade as alcohol withdrawal syndrome, and how antidepressant toxicity belongs on the differential in any patient presenting with unexplained encephalopathy and autonomic instability, particularly when a reliable history is unavailable. Early recognition of bupropion toxicity, even when confirmatory testing is pending, can guide appropriate cardiac monitoring and anticipatory seizure management, and help avoid unnecessary escalation of alcohol withdrawal-directed therapy.

## Introduction

Bupropion is a widely prescribed aminoketone antidepressant approved for the treatment of major depressive disorder, seasonal affective disorder, and smoking cessation [1]. It is also commonly used off-label for anxiety disorders, attention-deficit/hyperactivity disorder, bipolar disorder, and obesity [2]. Unlike selective serotonin reuptake inhibitors, bupropion inhibits the reuptake of norepinephrine and dopamine [2,3] and lowers the seizure threshold in a dose-dependent manner, with a recommended maximum daily dose of 450 mg/day for extended-release formulations [1]. Extended-release formulations carry an additional risk, as prolonged absorption and accumulation of the active metabolite, hydroxybupropion, can delay and prolong neurotoxicity [4,5].

The clinical picture of bupropion toxicity can include a range of neurologic and cardiovascular complications, including agitation, encephalopathy, hallucinations, hypertension, seizures, tremors, arrhythmias, and, in severe cases, cardiogenic shock [4]. Despite reformulation efforts and revised dosing recommendations over the years, overdose remains common. According to 2023 data from the National Poison Data System, more than 20,488 bupropion exposures were reported to United States Poison Control Centers, 10,868 of which involved single-substance ingestion [6]. Seizures occur in an estimated 17% to 47% of bupropion overdoses and are frequently preceded by non-specific symptoms such as agitation, acute encephalopathy, and tremors [4,7].

Because bupropion toxicity and alcohol withdrawal syndrome cause central nervous system hyperexcitability and sympathetic overactivity, distinguishing between them on clinical grounds alone can be difficult [8,9]. The difficulty is compounded when patients have a history of substance use disorder, a setting in which diagnostic anchoring toward alcohol withdrawal is understandable but potentially misleading. We present a case of bupropion toxicity that was managed as alcohol withdrawal syndrome throughout the entire hospital course, in which the true etiology was not established until quantitative serum bupropion and hydroxybupropion levels returned after discharge. This case adds to the limited literature in which bupropion toxicity was confirmed exclusively after discharge, and reinforces the need for clinicians to consider antidepressant toxicity as a primary etiology in patients presenting with undifferentiated encephalopathy and autonomic instability [4,5].

## Case presentation

A 55-year-old male presented to the emergency department (ED) with agitation and altered mental status following an episode of convulsions and loss of consciousness. His medical history was significant for type 2 diabetes mellitus, hypertension, major depressive disorder, and opioid use disorder, for which he was actively undergoing rehabilitation. At the time of presentation, the accompanying family member was unable to provide details regarding his medication-assisted treatment regimen. Chart review revealed home medications of bupropion XL (extended-release) 300 mg twice daily, a total daily dose exceeding the recommended maximum, losartan 50 mg daily, and metformin 1000 mg twice daily.

Approximately six hours before presentation, a family member observed the patient exit the bathroom and vomit six times. He then appeared to fall asleep and remained unarousable for approximately one hour. On awakening, he developed whole-body tremors, but was briefly communicative before his mental status deteriorated into increasing agitation and visual and auditory hallucinations. He arrived at the ED combative and diaphoretic, with a respiratory rate of 20 breaths per minute, a heart rate of 140 beats per minute, and systolic blood pressure in the 180s mmHg, and was oriented only to self, limiting the ability to obtain a reliable history.

**Figure 1:**
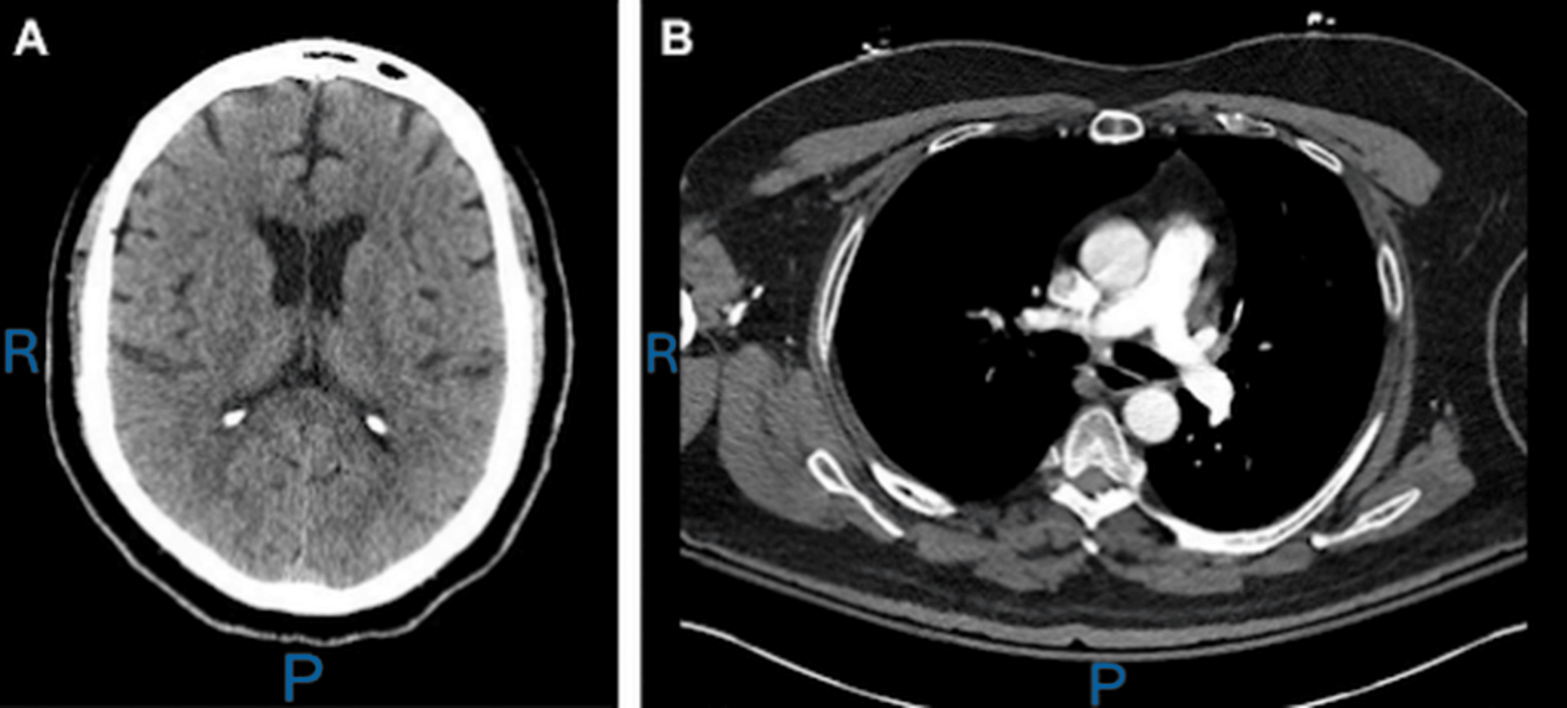
Radiologic imaging findings. (A) Axial computed tomography (CT) of the head demonstrates symmetric lateral ventricles with preserved gray-white differentiation and no evidence of acute intracranial hemorrhage, mass effect, or midline shift. (B) Axial CT pulmonary angiogram demonstrates normal contrast opacification of the main pulmonary artery and its right and left branches without evidence of pulmonary embolism. "R" denotes right-sided laterality and "P" denotes posterior direction.

**Figure 2:**
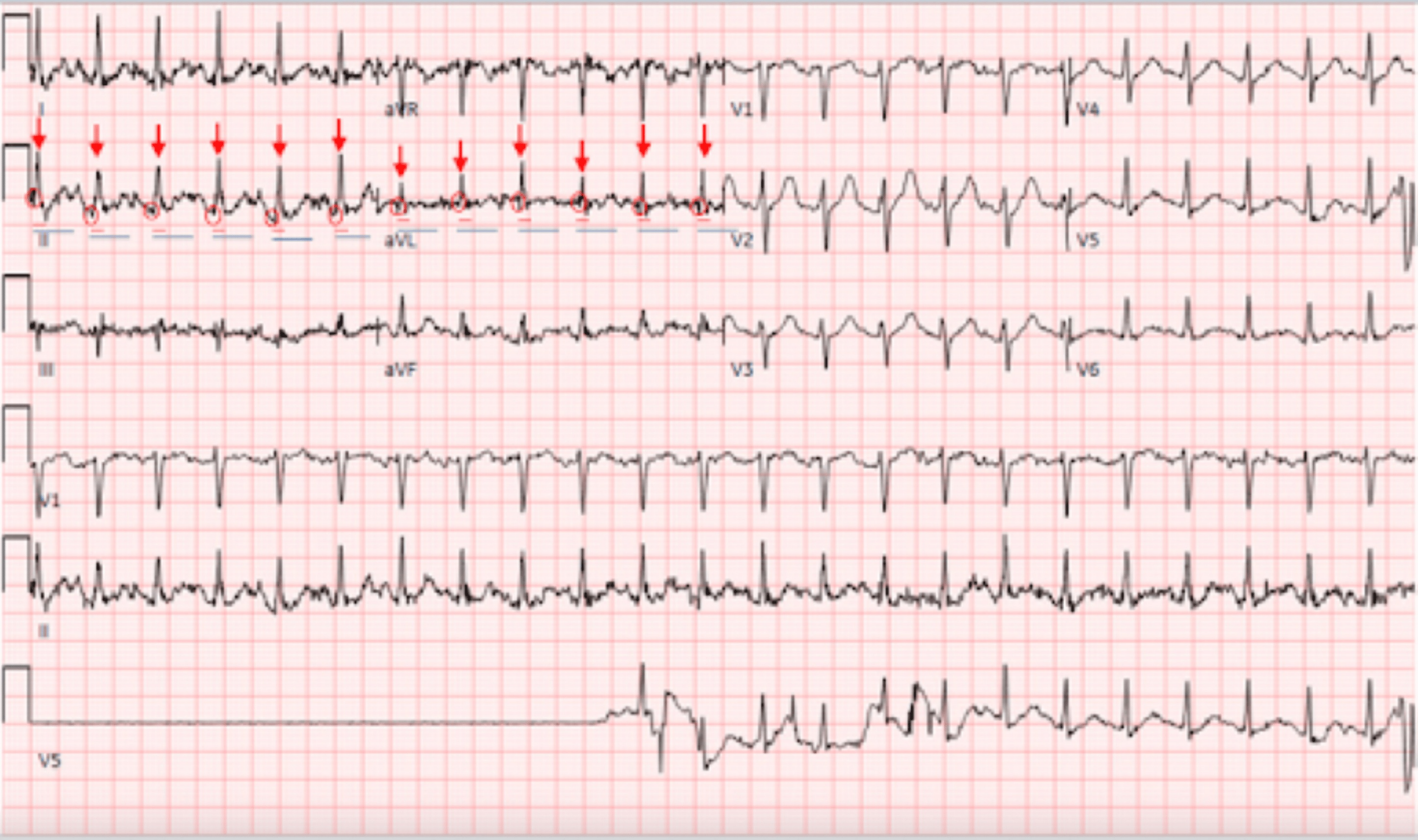
Electrocardiogram demonstrating sinus tachycardia without QRS widening or QT interval prolongation. In lead II, red circles highlight upright P-wave morphology, red arrows highlight narrow QRS complexes, red horizontal lines highlight QRS duration less than three small boxes (less than 120 milliseconds), and blue horizontal lines highlight QT intervals less than 10 small boxes (less than 400 milliseconds). Baseline artifact is present, likely related to patient agitation.

Initial laboratory studies revealed leukocytosis (white blood cell count: 15.9 x 10^3^/µL), elevated lactate (lactate: 6.3 mmol/L), an anion gap of 13 mEq/L, presumed acute kidney injury (creatinine: 1.36 mg/dL), hyperglycemia (glucose 253 mg/dL), and elevated creatine kinase (225 U/L) (Table 1). Noncontrast computed tomography (CT) images of the head and CT angiography of the chest were unremarkable (Figures 1A-1B, respectively). Electrocardiography demonstrated sinus tachycardia without QRS widening or QT interval prolongation (Figure 2). Serum ethanol, urine toxicology screen, and heavy metal testing were negative. The patient had no documented history of chronic heavy alcohol use, and collateral history from the accompanying family member did not suggest recent alcohol consumption prior to symptom onset. Other toxicologic syndromes, including sympathomimetic intoxication, serotonin syndrome, anticholinergic toxidrome, and opioid-related neurotoxicity, were included in the initial differential. However, a negative urine drug screen, along with the overall clinical picture, was not supportive of these diagnoses. Of note, methadone and tramadol-associated neurotoxicity were considered in the differential given his history of opioid use disorder and active participation in an outpatient rehabilitation program. However, the absence of classic opioid toxidrome features, including miosis and respiratory depression, made opioid-related neurotoxicity unlikely as a primary contributor.

**Table 1:** Key laboratory findings.

Test	Result	Reference Range	Interpretation
Laboratory Parameters			
White blood cell count	15.9 x 10^3^/µL	3.8-10.6 x 10^3^/µL	Elevated
Lactate	6.3 mmol/L	0.4 to <2.00 mmol/L	Elevated
Anion gap	13 mEq/L	8-16 mEq/L	Normal
Creatinine	1.36 mg/dL	0.7-1.4 mg/dL	Elevated
Glucose	253 mg/dL	70-110 mg/dL	Elevated
Creatine kinase	225 U/L	55-170 U/L	Elevated
Drug Levels			
Bupropion	133 ng/mL	Therapeutic range: 10-100 ng/mL, Toxic: Greater than or equal to 400 ng/mL	Elevated
Hydroxybupropion	>3,000 ng/mL	Therapeutic range: 850-1500 ng/mL, Toxic: Greater than or equal to 2000 ng/mL	Elevated
Metformin	1.7 µg/mL	Therapeutic range: ~1-2 µg/mL, Reporting limit: 0.10 µg/mL	Within therapeutic range
Methadone	303 ng/mL	No therapeutic range available. Positive cutoff: 10 ng/mL	Detected
2-ethylidene-1,5-dimethyl-3,3-diphenylpyrrolinium	39 ng/mL	No therapeutic range available. No positive cutoff available	Detected

Given the constellation of agitation, tremors, autonomic instability, suspected seizure activity, and history of substance use disorder, alcohol withdrawal syndrome was the working diagnosis. Elevated lactate and creatine kinase were interpreted as consistent with an unwitnessed seizure, further supporting this impression. The Clinical Institute Withdrawal Assessment for Alcohol-Revised (CIWA-Ar) protocol was initiated [10]; due to a score of 20 (Table 2), the patient received intravenous lorazepam and was admitted to the intensive care unit (ICU). The score was primarily driven by severe agitation, tremor, hallucinations, and impaired orientation.

**Table 2:** Clinical Institute Withdrawal Assessment for Alcohol-Revised assessment domains and range of scores for each domain.

Clinical Institute Withdrawal Assessment for Alcohol-Revised Scale	
Domains	Range of Scores
Nausea/vomiting	0-7
Tremor	0-7
Paroxysmal sweats	0-7
Anxiety	0-7
Agitation	0-7
Tactile disturbances	0-7
Auditory disturbances	0-7
Visual disturbances	0-7
Headache/fullness in head	0-7
Orientation/clouding of sensorium	0-4

**Figure 3:**
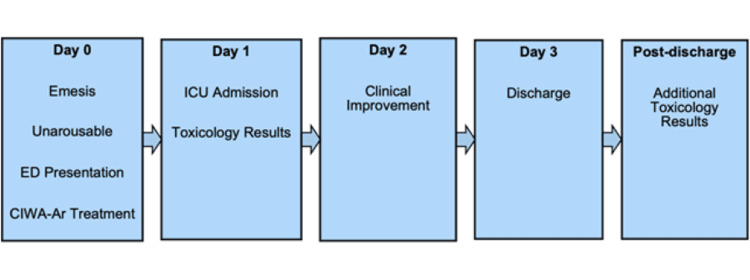
Timeline of the patient’s clinical course. ED: emergency department; CIWA-Ar: Clinical Institute Withdrawal Assessment for Alcohol-Revised Source: This figure was created by the authors using Microsoft Word (Microsoft Corp., Redmond, WA, USA).

Due to ongoing diagnostic uncertainty, a serum toxicology screen and quantitative serum levels of bupropion, hydroxybupropion, and metformin were sent shortly after ICU admission. Quantitative bupropion and hydroxybupropion levels were ordered specifically because the patient's prescription for supratherapeutic bupropion dosing had not been fully evaluated as a potential contributor to his presentation. Metformin levels were ordered, given elevated lactate in the setting of concurrent metformin use, to evaluate for metformin-associated lactic acidosis. During his hospital course, the patient was managed with supportive care and benzodiazepines. Collateral information obtained from the patient's wife on hospital day 2 confirmed that he was receiving methadone 90 mg daily as part of his outpatient rehabilitation regimen. No further seizures occurred. His elevated lactate, acute kidney injury, and mild rhabdomyolysis resolved with intravenous fluid resuscitation. Mental status gradually returned to baseline. A timeline summarizing the patient's clinical course is shown in Figure 3. After recovery, the patient denied recent alcohol use and denied intentional bupropion overdose.

The remaining toxicology results returned after discharge. Metformin returned within the therapeutic range at 1.7 µg/mL. However, bupropion was elevated at 133 ng/mL, and hydroxybupropion exceeded 3,000 ng/mL, surpassing the reporting laboratory's threshold for significant toxicity (Table 1). Methadone was detected at 303 ng/mL and its inactive metabolite, 2-ethylidene-1,5-dimethyl-3,3-diphenylpyrrolinium (EDDP), at 39 ng/mL (Table 1). A phosphatidylethanol (PEth) level drawn on admission also returned negative, excluding recent significant alcohol consumption. These results had no influence on real-time management decisions, a practical limitation inherent to quantitative toxicology testing that directly contributed to the diagnostic delay.

## Discussion

When this patient presented with agitation, tremor, and hemodynamic instability in the setting of a history of substance use disorder and an unreliable history, alcohol withdrawal was a reasonable initial working diagnosis. Furthermore, methadone was not identified as part of the patient's regimen until hospital day 2, limiting the team's ability to fully contextualize his opioid-related risk at the time of initial evaluation. The elevated lactate and creatine kinase suggested a recent seizure [11,12], and a CIWA-Ar score of 20 met criteria for severe withdrawal [10]. Metformin-associated lactic acidosis was also considered, given an elevated lactate level and concurrent metformin use. However, the metformin level returned within the therapeutic range at 1.7 µg/mL, the anion gap was within normal limits at 13 mEq/L, and the lactate normalized promptly with intravenous fluid resuscitation, a pattern more consistent with type B lactic acidosis from seizure-related metabolic demand than with metformin toxicity [11].

Importantly, the absence of detectable ethanol on initial testing did not exclude withdrawal, as serum ethanol levels may normalize before the onset or peak of symptoms in chronically dependent individuals [9]. In this case, the negative ethanol was not interpreted in isolation. A negative PEth, a direct biomarker of cumulative alcohol intake detectable for up to four weeks after consumption [13], combined with the patient's denial of recent alcohol use and the absence of a documented history of chronic heavy alcohol dependence, collectively provided a stronger basis for deprioritizing alcohol withdrawal than any single negative test alone. Although methadone was subsequently detected at a concentration consistent with his maintenance dose of 90 mg daily [14], and detectable EDDP was consistent with ongoing methadone metabolism rather than acute ingestion [15], the absence of opioid toxidrome features throughout the clinical course made acute methadone toxicity unlikely as a primary contributor to the presentation. Nevertheless, the true etiology remained elusive throughout the hospital course and only became apparent after discharge, when quantitative bupropion and hydroxybupropion levels returned markedly elevated.

Bupropion lowers the seizure threshold through dopaminergic and noradrenergic activity and inhibition of neuronal gap junctions [5]. Its primary active metabolite, hydroxybupropion, has a longer elimination half-life of up to 32 hours and may correlate more closely with neurologic toxicity than the parent compound [4,5]. Hydroxybupropion concentrations often exceed those of bupropion and persist well after parent drug levels begin to decline, particularly with extended-release formulations [1,4,5,16]. The dose-dependent nature of bupropion's seizure risk is also relevant here, as this patient was prescribed bupropion XL for major depressive disorder at 600 mg/day, exceeding the recommended maximum of 450 mg/day; the duration of therapy at this dose could not be confirmed from the available records, though it may have contributed to the severity of his presentation [1,5]. The bupropion XL prescribing information further identifies a history of substance use disorder and abrupt alcohol discontinuation as factors that may further increase seizure risk [1]. Although alcohol use was not laboratory-confirmed in this patient, his history of opioid use disorder may have represented an additional clinical factor contributing to his overall seizure risk [1]. Active methadone maintenance introduced a layer of pharmacologic complexity that, while not causally implicated, could not be entirely excluded as a contributing variable. Collectively, these pharmacokinetic and pharmacodynamic factors help explain the unpredictable and sometimes prolonged clinical course of bupropion toxicity and are clinically relevant in determining the appropriate duration of monitoring. Given the risk of delayed or recurrent seizures with extended-release formulations, a minimum observation period of 24 hours is recommended following suspected bupropion XL overdose [4,17].

The hydroxybupropion concentration exceeded the assay's upper reporting limit (>3,000 ng/mL), and the reporting laboratory defined toxic thresholds of 400 ng/mL or greater for bupropion and 2,000 ng/mL or greater for hydroxybupropion (Table 1). Although the bupropion level of 133 ng/mL remained below the toxic threshold, hydroxybupropion accumulation was well into the toxic range, with a true concentration that cannot be determined from these results alone. Several factors are relevant to interpreting these levels in context. Blood was drawn at a time that may not have reflected peak concentrations; bupropion undergoes extensive first-pass hepatic metabolism with rapid conversion to hydroxybupropion, which may account for the parent drug level remaining below the toxic threshold despite clear clinical toxicity [1,5]; and the extended-release formulation may have sustained ongoing absorption beyond the time of sampling [1,5]. Although bupropion is a potent inhibitor of CYP2D6, which can affect the metabolism of co-administered substrates, the patient's current medications, losartan and metformin, are not significantly metabolized by this pathway, making clinically meaningful CYP2D6-mediated drug interactions unlikely [5]. Bupropion is primarily metabolized to hydroxybupropion via CYP2B6 [5]. In this case, the combination of supratherapeutic dosing, extended-release formulation kinetics, and individual variability in CYP2B6 activity likely contributed to the degree of hydroxybupropion accumulation observed, and collectively illustrates why quantitative drug levels must be interpreted alongside timing of ingestion, formulation type, and individual metabolic factors rather than in isolation [5].

Bupropion toxicity and alcohol withdrawal syndrome share several overlapping clinical features that can complicate bedside diagnosis (Table 3). Both conditions are characterized by tremor, agitation, hallucinations, seizures, central nervous system hyperexcitability, and sympathetic overactivity [8,9]. However, their underlying pathophysiology differs. Alcohol withdrawal reflects decreased γ-aminobutyric acid (GABA) activity and upregulation of N-methyl-D-aspartate (NMDA) receptor activity following chronic alcohol exposure, typically following a predictable timeline beginning six to 24 hours after cessation [8,9]. In contrast, bupropion toxicity results from excess catecholaminergic activity due to norepinephrine and dopamine reuptake inhibition, with a clinical course that is considerably less predictable, particularly with extended-release formulations [4,5,16]. In our assessment, clinical features that should prompt reconsideration of alcohol withdrawal include lack of objective evidence of recent alcohol use, a timeline inconsistent with expected withdrawal progression, delayed or recurrent seizures, and a medication list that includes bupropion at or above recommended doses.

**Table 3:** Neurotransmitter disruptions in alcohol withdrawal syndrome and bupropion toxicity.

Feature	Alcohol Withdrawal Syndrome	Bupropion Toxicity
Primary neurotransmitter disruption	↓ γ-aminobutyric acid activity	↑ Dopamine and norepinephrine
Excitatory pathway	↑ N-methyl-D-aspartate receptor activity	Catecholamine accumulation from norepinephrine transporter and dopamine transporter inhibition
Resulting neurophysiology	Central nervous system hyperexcitability due to loss of inhibitory tone	Increased catecholaminergic stimulation
Clinical overlap	Tremor, agitation, hallucinations, seizures	Tremor, agitation, hallucinations, seizures

Management of bupropion overdose is mainly supportive. Initial priorities include stabilization of airway, breathing, and circulation [5,16]. Activated charcoal may be considered within one hour of ingestion in patients who are alert, not actively seizing, and able to protect their airway [18]. Whole bowel irrigation may have a role in large ingestions involving extended-release formulations [19]. Intravenous benzodiazepines are the recommended first-line treatment for bupropion-associated seizures and are also effective for the agitation, tachycardia, and tremors that frequently precede seizure activity [4,17]. Continuous cardiac monitoring is essential due to the risk of life-threatening arrhythmias, with attention to QRS and QT interval prolongation [4,7]. In cases of severe cardiovascular collapse, intravenous lipid emulsion therapy and extracorporeal membrane oxygenation have been used [20].

An important practical consideration is that benzodiazepines are appropriate initial treatment for both alcohol withdrawal and bupropion toxicity. As a result, misdiagnosis may not immediately result in inappropriate treatment, but may eventually lead to insufficient cardiac monitoring, underestimation of the risk for delayed seizures, and unnecessary escalation of alcohol withdrawal-directed therapies. Although quantitative serum bupropion and hydroxybupropion levels are not routinely required for management decisions, they can provide meaningful diagnostic confirmation in cases with an ambiguous clinical presentation [4,5].

This case has several limitations. The exact quantity of bupropion ingested is unknown, as the patient denied intentional overdose after recovery, and no pill count was available. Whether the supratherapeutic prescribed dose of bupropion represented a prescribing error or intentional off-label dosing could not be determined [1,5]. Although alternative etiologies were thoroughly evaluated, including negative ethanol testing, comprehensive toxicology screening, negative PEth, and normal neuroimaging, several uncertainties remain. Post-discharge methadone levels were consistent with his confirmed maintenance dose, and detectable EDDP supported ongoing metabolism rather than acute ingestion [15]. However, whether methadone maintenance affected overall seizure susceptibility could not be determined from the available data. An unwitnessed co-ingestion of another substance cannot be entirely excluded, and while negative ethanol testing, negative PEth, and patient denial collectively argued against significant recent alcohol use, alcohol withdrawal cannot be entirely excluded. Finally, although both bupropion and hydroxybupropion concentrations were elevated, the bupropion level of 133 ng/mL remained well below concentrations reported in fatal and near-fatal intoxications, while the hydroxybupropion level exceeded the upper reporting limit of the assay at >3,000 ng/mL, limiting the ability to directly correlate these levels with clinical severity [5].

## Conclusions

Bupropion toxicity and alcohol withdrawal syndrome can present in ways that are difficult to distinguish, particularly in patients with substance use histories and limited capacity to provide history at the time of evaluation. In the case presented here, an entirely reasonable initial diagnosis of alcohol withdrawal delayed recognition of the true toxicologic etiology until after discharge. Fortunately, management with benzodiazepines and supportive care is appropriate for both conditions, and the patient recovered fully. The more important lesson is one of monitoring and anticipation, as early consideration of bupropion toxicity prompts appropriate cardiac surveillance, a lower threshold for prolonged observation given the risk of delayed seizures, and avoidance of unnecessary escalation of alcohol withdrawal-specific therapies. This vigilance is especially warranted in patients with compounding risk factors such as supratherapeutic dosing, substance use disorder, or concurrent medications that may further lower the seizure threshold. In any patient on bupropion who presents with unexplained encephalopathy, autonomic instability, or seizures, quantitative bupropion and hydroxybupropion levels are worth obtaining early, even if the clinical picture initially suggests another diagnosis. Whether this testing should be incorporated more routinely into the workup of undifferentiated toxic presentations in patients on psychiatric medications is a question worth exploring prospectively.
